# Social Isolation During COVID-19 Pandemic. Perceived Stress and Containment Measures Compliance Among Polish and Italian Residents

**DOI:** 10.3389/fpsyg.2021.673514

**Published:** 2021-05-28

**Authors:** Jakub Grabowski, Joanna Stepien, Przemyslaw Waszak, Tomasz Michalski, Roberta Meloni, Maja Grabkowska, Aleksandra Macul, Jakub Rojek, Liliana Lorettu, Iwona Sagan, Leszek Bidzan

**Affiliations:** ^1^Department of Developmental Psychiatry, Psychotic and Geriatric Disorders, Medical University of Gdańsk, Gdańsk, Poland; ^2^Department of Socio-Economic Geography, Faculty of Oceanography and Geography, University of Gdańsk, Gdańsk, Poland; ^3^Department of Hygiene and Epidemiology, Medical University of Gdańsk, Gdańsk, Poland; ^4^Department of Regional Development, Faculty of Oceanography and Geography, University of Gdańsk, Gdańsk, Poland; ^5^University of Sassari, Sassari, Italy; ^6^Adult Psychiatry Scientific Circle, Department of Developmental Psychiatry, Psychotic and Geriatric Disorders, Medical University of Gdańsk, Gdańsk, Poland; ^7^Psychiatric Clinic, Department of Medical, Surgical and Experimental Sciences, University of Sassari, Sassari, Italy

**Keywords:** SARS-CoV-2, quarantine, spatial mobility, guideline adherence, physical activity, social behavior, physical distancing, mental disorders

## Abstract

**Background:**

In this study, we analyze the association of social isolation in the first phase of the pandemic with perceived stress among residents of Poland and Italy with a look at how these populations adjust to and comply with implemented regulations, guidelines, and restrictions.

**Materials and Methods:**

Internet survey with Perceived Stress Scale (PSS-10) and questions regarding mobility patterns, attitude, and propensity to adjust toward the implemented measures and current health condition was made among Polish and Italian residents (Cronbach’s alpha 0.86 and 0.79, respectively). The sample size was 7,108 (6,169 completed questionnaires in Poland and 939 in Italy).

**Results:**

The Polish group had a higher stress level than the Italian group (mean PSS-10 total score 22,14 vs 17,01, respectively; *p* < 0.01). There was a greater prevalence of chronic diseases among Polish respondents. Italian subjects expressed more concern about their health, as well as about their future employment. Italian subjects did not comply with suggested restrictions as much as Polish subjects and were less eager to restrain from their usual activities (social, physical, and religious), which were more often perceived as “most needed matters” in Italian than in Polish residents.

**Conclusion:**

Higher activity level was found to be correlated with lower perceived stress, but the causality is unclear. Difference in adherence to restrictions between Polish and Italian residents suggests that introducing similar lockdown policies worldwide may not be as beneficial as expected. However, due to the applied method of convenience sampling and uneven study groups, one should be careful with generalizing these results.

## Introduction

During the early months of 2020, the first coronavirus disease 2019 (COVID-19) infections were recorded in all European countries with Europe being considered by the WHO as the active center of the COVID-19 pandemic.^1^ Following a rapid increase of new cases and deaths, preventive measures to mitigate the pandemic were taken *ad hoc* by all countries. These measures often included the imposition of lockdowns with restrictions varying across the continent. As of 18th March, more than 250 million people were in lockdown in Europe.^2^

Prolonged social isolation is known to be associated with increased morbidity and mortality in the human population. In terms of mental health, it leads to depression, anxiety, suicidality, personality disorders, psychoses, and deterioration of cognitive functions (Cacioppo et al., 2011; Wang et al., 2017; Zorzo et al., 2019; Chu et al., 2020). Recent studies confirm that current long-term health threats and imposed deprivation of social contacts associated with a feeling of lack of control lead to a significant increase in mental health issues in the global population (Gobbi et al., 2020). Other risk factors for the deterioration of mental condition involve female gender, younger age, and lack of approval for the policy of the country toward the COVID-19 pandemic. Insomnia—as a possible prodrome for anxiety and depressive disorders—was the most commonly recorded issue.

Pandemic Management Theory (PMT), developed by Stueck (2021), suggests a complex mechanism behind the mental health burden pointing to a possible role of several fears in impacting individual and collective coping processes. Fears of losing autonomy, getting sick, losing energy, aggression by others, or fear of the future are postulated to significantly exceed the actual fear of death, which is contrary to what is observed in individuals with an experience of trauma caused by a terror attack. Deprivation of physical or eye contact and restricted field of vision due to mask-wearing are also suggested to lead to empathy and emotional dysregulation (Stueck, 2021). According to PMT, difficulty in sustaining biocentric connections of individuals to themselves, others, and nature during the pandemic may play a crucial role in the development of anxiety, depression, stress, and post-traumatic stress, loneliness, social isolation, and stigma. Similar observations were made by the study of Super et al. (2020) where mental health condition was predicted by the sense of individual and national coherence and the presence of social support.

Only small differences in the proportions of psychiatric outpatients reporting worsening of their mental condition were observed between European countries (e.g., 53.72% for Poland and 48.86% for Italy) (Gobbi et al., 2020). This remains not fully understandable, as the course of the pandemic, the socioeconomic or cultural backgrounds, and the imposed restrictions across Europe varied significantly between the countries and should therefore lead to observed differences in the level of distress. Several studies show that the overall functioning deterioration rates and experienced stress levels during the COVID-19 outbreak are very high across the general population (Gualano et al., 2020; Pakenham et al., 2020; Pieh et al., 2020; Ares et al., 2021; Tan et al., 2021), and are associated, among others, with the loss of job and social support, fear of infection, change of lifestyle, and isolation (Dymecka et al., 2020). Still, little is known on predispositions to mental health deterioration and the presence of any possible protective factors. Furthermore, the lack of comparative cross-country studies evaluating stress and lockdown compliance (Castex et al., 2020) makes it difficult to draw reliable conclusions on the positive and negative effects of the imposed restrictions.

Considering the aforementioned differences across countries and their inhabitants, it seems that introducing similar or identical policies across different regions of the world may not be as beneficial as initially expected, with varied short- and long-term consequences for physical and mental health. Furthermore, “flattening the curve” will not be achievable if the introduced regulations are met with low compliance due to low social acceptability. In our study, we compare the association of social isolation in the first phase of the COVID-19 pandemic with perceived stress among residents of Poland and Italy with a look at how these populations adjust to and comply with implemented regulations, guidelines, and restrictions. These two countries were chosen for analysis due to cultural differences and a significant contrast in the severity of the first wave of pandemic (Supplementary Figures 1, 2) (Dong et al., 2020; Roser et al., 2020), to verify whether the above-mentioned reasons impacted compliance to introduced lockdown policies. Furthermore, the survey was meant to give an initial look at the possible association of applied restrictions (similar in Poland and Italy) leading to social isolation with increased stress perception in different sociocultural groups.

## Materials and Methods

### Participants and Procedure

Being a part of a larger analysis on spatial mobility, this study was based on an internet survey made between adult Polish and Italian residents during the first wave of the COVID-19 pandemic (April–May 2020). The distribution of the survey was made through the national and local media, websites of individual regions and provincial cities, social media, and university newsletters with the goal of achieving country-wide responses. It also partially relied on virtual snowball sampling. Participants provided electronic informed consent prior to the survey and were free to quit it at any time. This study complied with all the ethical guidelines and standards for online surveys with human participants, in accordance with the local legislations. The study was conducted in accordance with the ethical principles of the Declaration of Helsinki.

The sample size was 7,108 with 6,169 completed questionnaires in Poland (77% females vs. 23% males) and 939 in Italy (62.8% females vs. 37.1% males). Detailed sociodemographic data can be found in Table 1. We have gathered information only on the country of residence, not on the nationality of respondents, as the basis of the study was to analyze the spatial mobility changes as a reaction to the implementation of governmental restrictions and the course of the pandemic. While COVID-19 infection and lethality rates differed greatly between the two countries in the aforementioned period (Supplementary Figures 1, 2) (Dong et al., 2020; Roser et al., 2020), the applied lockdown regulations were similar. At the time of the survey, the following restrictions were in force in both countries: closures (all public and private educational institutions, shopping malls, public events, entertainment and recreation facilities, and cultural institutions), limitations (public gatherings, public transport, restaurants, and travel), and warrants (social distance, civil quarantine, covering of mouth and nose, border closures, and controls). Detailed chronological information on implemented rules and regulations in Poland and Italy can be found in the Supplementary material [Supplementary Table 1 based on Pinkas et al. (2020) and updated by authors of this manuscript; Supplementary Table 2] (Governo Italiano Presidenza del Consiglio dei Ministri, 2021).

**TABLE 1:** Sociodemographic characteristics of the respondents.

	**Polish residents (*N* = 6,169)**	**PSS score (mean) ± SD**	**Italian residents (*N* = 939)**	**PSS score (mean) ± SD**	***P*-value**
**Sex**					
Female	4698 (77%)	22,88 ± 7,37	587 (62,8%)	18,10 ± 7,37	< 0,0001
Male	1402 (23%)	19,66 ± 7,69	347 (37,1%)	15,23 ± 7,69	< 0,0001
**Age**					
18–24	1945 (31,5%)	23,32 ± 7,64	91 (9,7%)	20,65 ± 7,07	< 0,0001
25–34	2034 (33%)	21,95 ± 7,72	243 (25,9%)	18,00 ± 6,75	< 0,0001
35–44	1270 (20,6%)	21,45 ± 7,47	203 (21,6%)	16,80 ± 7,72	< 0,0001
45–54	552 (8,9%)	21,35 ± 7,19	184 (19,6%)	15,88 ± 6,52	< 0,0001
55–64	259 (4,2%)	20,76 ± 6,27	180 (19,2%)	15,52 ± 6,36	< 0,0001
65–74	95 (1,5%)	19,99 ± 6,37	38 (4%)	14,97 ± 6,77	< 0,0001
75 +	14 (0,2%)	19,93 ± 9,04	0	N/A	
**Education**					
Higher	4194 (68%)	21,72 ± 7,54	599 (63,4%)	16,74 ± 6,9	< 0,0001
Secondary	1821 (29,5%)	23,13 ± 7,60	290 (30,7%)	17,56 ± 7,16	< 0,0001
Vocational	65 (1%)	20,32 ± 7,27	21 (2,2%)	13,81 ± 7,22	< 0,01
Junior high school	76 (1,2%)	23,66 ± 7,02	34 (3,6%)	19,09 ± 7,64	< 0,01
Primary	13 (0,2%)	20,31 ± 5,12	0		
**Marital status**					
Single	2690 (43,6%)	22,42 ± 7,67	266 (28,2%)	18,20 ± 7,07	< 0,0001
Married/long-term relationship	3166 (51,3%)	21,92 ± 7,51	613 (65%)	16,62 ± 7,01	< 0,0001
Divorced	255 (4,1%)	22,03 ± 7,40	49 (5,2%)	15,47 ± 6,73	< 0,0001
Widowed	58 (0,9%)	21,86 ± 6,72	15 (1,6%)	17,13 ± 6,86	< 0,05
**Employment status**					
Employment contract	2943 (51,2%)	21,43	472 (64,1%)	16,35 ± 7,02	< 0,0001
Mandate contract	357 (6,2%)	23,34	11 (1,5%)	16,36 ± 9,31	< 0,05
Self-employment	477 (8,3%)	21,65	0	N/A	
Pension	149 (2,6%)	20,81	50 (6,8%)	15,38 ± 6,28	< 0,0001
Student	1630 (28,3%)	23,17	140 (19,0%)	20,06 ± 7,01	< 0,0001
Unemployed	197 (3,4%)	23,89	63 (8,6%)	19,03 ± 6,96	< 0,0001
**Type of settlement**					
City over 500,000 residents	2249 (36,5%)	21,91 ± 7,81	92 (9,8%)	17,23 ± 7,07	< 0,0001
City 150,000–500,000 residents	1894 (30,7%)	21,89 ± 7,48	96 (10,2%)	17,65 ± 7,43	< 0,0001
Town 50,000–150,000 residents	604 (9,8%)	23,06 ± 7,42	385 (41%)	17,37 ± 6,67	< 0,0001
Town under 50,000 residents	730 (11,8%)	22,64 ± 7,54	150 (16%)	16,03 ± 6,96	< 0,0001
Village/rural area	692 (11,2%)	22,24 ± 7,12	217 (23,1%)	16,69 ± 7,55	< 0,0001
**Number of people in household**					
1	664 (10,8%)	21,58 ± 7,83	132 (14,1%)	16,84 ± 7,08	< 0,0001
2	1998 (32,4%)	21,51 ± 7,74	259 (27,6%)	16,2 ± 7,05	< 0,0001
3	1473 (23,9%)	22,43 ± 7,42	239 (25,5%)	17,52 ± 6,85	< 0,0001
4	1409 (22,8%)	22,91 ± 7,41	241 (25,7%)	17,17 ± 7,24	< 0,0001
5 and more	625 (10,1%)	22,34 ± 7,26	67 (7,1%)	18,28 ± 7,26	0, 0002
**Work during pandemic**					
Work from home	2582 (41,8%)	21,38 ± 7,38	424 (45,1%)	16,93 ± 7,03	< 0,0001
Work from office (as before)	931 (15,1%)	21,16 ± 7,60	143 (15,2%)	14,71 ± 6,71	< 0,0001
Work suspended	932 (15,1%)	23,63 ± 7,62	151 (16%)	18,3 ± 6,58	< 0,0001
Not applicable	1724 (27,9%)	23,00 ± 7,59	223 (23,7%)	17,78 ± 7,28	< 0,0001
**Household financial situation**					
Live very well	1543 (25,0%)	20,32 ± (7,93)	117 (12,4%)	16,12 ± 6,76	< 0,0001
Doing fine	3589 (58,2%)	22,22 ± (7,32)	599 (63,6%)	16,71 ± 6,87	< 0,01
Hardly manage	716 (11,6%)	24,88 ± (6,85)	163 (17,3%)	18,98 ± 7,53	< 0,0001
Cannot handle this situation	134 (2,2%)	26,81 ± (7,21)	19 (2,0%)	18,21 ± 9,08	< 0,0001
**Self-assessed health status**					
Very good	2435 (39,5%)	19,66 ± (7,86)	271 (28,7%)	14,18 ± 7,1	< 0,0001
Good	2911 (47,2%)	22,85 ± (6,82)	471 (49,9%)	17,32 ± 6,51	< 0,01
Average	719 (11,7%)	26,73 ± (6,01)	160 (17,0%)	19,69 ± 6,14	< 0,01
Bad	87 (1,4%)	28,7 ± (6,87)	34 (3,6%)	20,97 ± 7,68	< 0,0001
Very bad	17 (0,3%)	29,59 ± (9,98)	7 (0,7%)	25,71 ± 11,61	0, 29

### Measures

The study consisted of three main parts. The first part was a sociodemographic questionnaire, including questions on current health condition and financial situation (items and possible answers can be found in Table 1). The second part was based on a questionnaire with five possible Likert-type scale responses (Likert, 1932). For respondents’ mobility patterns, attitude, and propensity to adjust toward the implemented measures and fears regarding health and economics, possible answers included “definitely agree,” “mostly agree,” “neither agree, nor disagree,” “mostly disagree,” and “definitely disagree.” Pessimism and optimism as traits were self-assessed using 1–5 scale, where 1 was described as pessimistic and 5 as optimistic. Questions regarding current respiratory tract infections and current or past COVID-19 infections in respondents and their close ones had possible “yes” and “no” answers. The final part of the survey was the administration of the Perceived Stress Scale (PSS-10) (Cohen et al., 1983) in its standard timespan version with the scoring of items 4, 5, 7, and 8 reversed. Polish and Italian translations were used.

### Statistical Strategy

Survey data were collected online using Google Forms (Google Inc., United States) and subsequently exported to Excel spreadsheets (Microsoft, United States). Statistical analysis was performed using STATISTICA 10.0 software (StatSoft Inc., United States). All of the quantitative variables were tested using Kolmogorov–Smirnov test, for meeting the criteria of a normal distribution (Gaussian distribution). Depending on whether the variable met the normality condition, appropriate statistical tests were applied at further stages. For comparisons between two groups, the parametric *t*-test or non-parametric Mann–Whitney *U*-test was used. For Gaussian data, comparing several groups, we used the one-way ANOVA. If the result was significant, for particular group differences, we ran *post hoc* Scheffé’s test (to minimize the potential unequal sample size bias). For comparing qualitative survey data, Pearson’s chi-squared test was used along with the calculation of observed frequencies (with appropriate Yates’ correction for small observed frequencies when necessary).

Reliability calculations of the PSS-10 questionnaire were made using statistical software MedCalc, version 15.8 (MedCalc Software Bvba, Ostend, Belgium). We calculated Cronbach’s alpha with raw variables along with the correction tool for scale reversal.

The statistically significant threshold level in all calculations was set at *p* < 0.05.

## Results

Sociodemographic data of Polish and Italian respondents, including several significant differences between study groups, are shown in Table 1. The percentages apply to the total number of participants who answered a specific question, not the whole study group (questions could be left unanswered).

Both language versions of PSS-10 questionnaires had a similar reliability coefficient. Cronbach’s alpha for the Polish version of the PSS-10 questionnaire was 0.86 and for the Italian version 0.79.

Results show higher stress levels in the Polish group than in the Italian responders with a mean PSS total score of 22,14 vs. 17,01, respectively, and *p* < 0.01 (Table 2). This significant distinction also applies to all sociodemographic subgroups (Table 1) and all items of PSS-10 except for item 4 regarding confidence about one’s ability to handle personal problems (Table 2). The highest stress level was observed in females, younger people, single, with an intermediate level of education (junior high school or secondary), living in larger households, with work suspended due to pandemic, and those assessing their health or financial situation negatively.

**TABLE 2:** Perceived Stress Scale (PSS-10) scores in Polish and Italian respondents.

	**Polish residents (*N* (=6169)**		**Italian residents (N (=939)**		***P*-value**
	**mean**	**SD**	**Mean**	**SD**	
PSS total score	22,14	7,57	17,01	7,04	<0,01
**PSS item**					
1. Been upset	2,23	1,08	1,61	1,06	<0,01
2. Unable to control	2,09	1,26	1,43	1,25	<0,01
3. Nervous-stressed	2,60	1,05	1,48	1,20	<0,01
4. Felt confident (R)	1,92	1,20	1,86	1,19	0,05
5. Things your way (R)	2,58	0,92	2,14	1,05	<0,01
6. Could not cope	1,77	1,24	0,98	1,21	<0,01
7. Control irritations (R)	2,21	1,11	2,01	1,21	<0,01
8. On top of things (R)	2,70	0,93	2,77	0,91	<0,05
9. Been angered	2,23	1,33	1,50	1,29	<0,01
10. Could not overcome	1,82	1,27	1,40	1,26	<0,01

**FIGURE 1:**
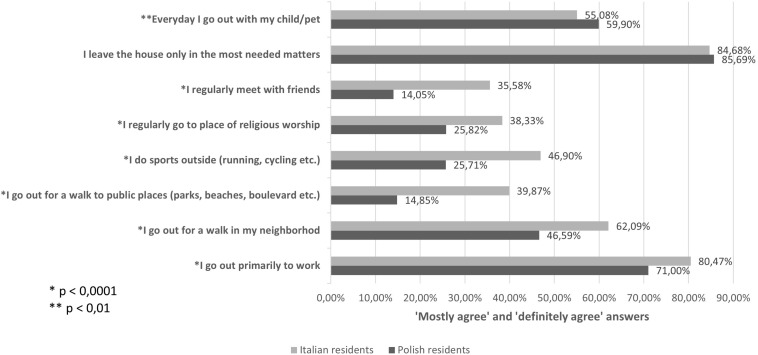
Pandemic restriction compliance.

**FIGURE 2:**
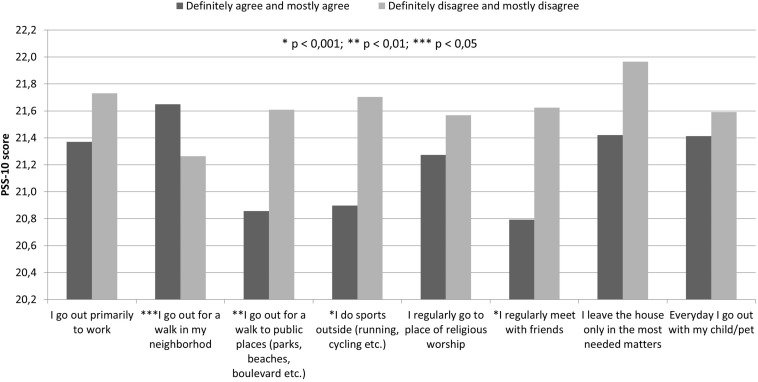
Perceived stress and restriction compliance.

In Figure 1, we present significant differences in complying with restrictions implemented by both governments. Since a major disparity between the countries was observed, showing much higher levels of compliance with restrictions among the Polish respondents, the data were double-checked for survey translation and statistical errors. For all presented specific activity types, *p*-value was estimated to be lower than 0.01. There were no significant differences in how the respondents self-assessed their need to leave the house. Compliance with restrictions and, consequently, lower declared activity were generally associated with higher PSS scores in the whole study group (Figure 2).

Other results show a greater prevalence of chronic diseases among Polish respondents (34.79 vs. 19.39%, *p* < 0.0001). Italians, on the other hand, more frequently expressed concern about their own health (64.15 vs. 53.38% Polish residents, *p* < 0.0001), as well as about their future employment (68.91 vs. 55.14%, *p* < 0.0001). Respondents diagnosed with a chronic disease more frequently expressed concern about their own health than those without such condition (67.75 vs. 48.49%, *p* < 0.0001). Similar attitudes were observed in answers regarding worries about the health of significant others (91.51% Italians vs. 90.55% Poles, *p* = 0.30), financial stability (74.23 vs. 75.78% consequently, *p* = 0.30), economic situation in the country of residence (94.69 vs. 94.09%, *p* = 0.47), and global economy (88.42 vs. 88.94%, *p* = 0.63). In self-assessment of personality traits, 55.19% Italian residents and 50.5% Polish residents described themselves as prevalently optimistic (*p* < 0.05). Only a small number of respondents admitted to having been diagnosed with COVID-19 (0.21% in the whole study group), having respiratory tract infection symptoms (6.02%), or having a close relative with current respiratory tract infection (6.14%), while 1.87% of the subjects confirmed the COVID-19 infection in their relatives.

## Discussion

To our best knowledge, this is the first study based on a relatively large sample that compares stress level and lockdown compliance between different countries. The reported perceived stress level measured by PSS-10 scale was significantly higher for Polish than for Italian residents but remained above average in both groups during the first wave of the COVID-19 pandemic. This tendency was observed among all sociodemographic subgroups. Compliance to lockdown restrictions was significantly higher among respondents based in Poland in comparison with those living in Italy despite the much more severe course of the first pandemic wave in the latter country.

We observed a relatively higher prevalence of chronic diseases in Polish residents, which could be a possible explanation of the obtained results, as the presence of somatic illness was associated with the actual level of worrying about one’s health. On the other hand, Italian residents were those who actually expressed more concern about their health or employment with no significant differences regarding other possible sources of distress between the two groups. Furthermore, as mentioned before, Italy faced a much more extreme situation with several new cases of disease and deaths reported every day in dramatic media coverage, which could also play a role in aggravating perceived stress (Wheaton et al., 2021). One possible explanation for observed distinctions may be that during the study, residents of both countries were in various phases of adaptation according to PMT (Stueck, 2021) when actual fear of death in people in Italy (rather than hypothetical at that time in Poland) switched the survival instinct into a mental defense strategy (Becker, 2021). Such fear may impact one’s perception of restriction oppressiveness, making them seem more acceptable (Dymecka et al., 2020) and consequently causing less distress.

Major differences were also observed between the two countries with regard to lockdown policy compliance. Results show a greater overall activity (physical, social, religious) and lower compliance among Italian residents compared with polish residents during the first wave of the pandemic. At the same time, both groups perceived their pursued activity as ‘‘most needed matters.’’ This distinction between views of social, religious, and health needs suggests possible personality trait-related differences between inhabitants of countries. The contrast in outdoor activity may also be partly explained by different weather conditions during the spring when the study was conducted. Higher temperatures in Italy compared with those in Poland, together with cultural factors and differences between countries in ways of spending time outside homes, could make it more difficult to keep Italians indoors in April and May 2020. According to Google mobility trends,^3^ a decrease in spatial mobility was observed in both countries during the first wave of COVID-19 pandemic. The decrease was much deeper in Italy and lasted longer than in Poland. While it may seem contradictory to our findings, several limitations of assessing mobility by mobile phone data were raised, including limited data in internet-enabled or low cell-tower-density areas and selection bias (Grantz et al., 2020). The former may be a significant issue in our sample, considering differences in types of settlement between Polish and Italian groups. Furthermore, the Google mobility data are not directly comparable between countries with a difference in the base level of activity in starting point in each country. If in Italy the spatial mobility was higher than in Poland before March of 2020, the drop in mobility may have been deeper during the first weeks of lockdown restrictions in relative values, even if absolute values were similar or lower.

Joint data for residents of both countries show that higher compliance is associated with higher levels of stress (Figure 2), but the causality is unclear. Increased stress with fear of contagion may prevent people from leaving home. On the other hand, imposed restrictions, closures of workplaces, recreation grounds, and forced social isolation are known to increase stress levels, for instance, as a result of deprivation of physical closeness (Stueck, 2021) or by limiting physical activity (López-Valenciano et al., 2021; Ruiz et al., 2021). With possible varying personality traits between residents of both countries (McCrae et al., 2005; Schmitt et al., 2007) and, consequently, different attitudes toward pandemic and lockdown policies (Schaller and Murray, 2008; Barceló, 2017), this may be one of the reasons behind perceived stress-level differences between the two discussed countries, although the actually imposed restrictions were similar in Poland and Italy at that time (Supplementary Tables 1, 2). One should also consider the possible impact of differences in the level of citizen trust in government between the two countries (OECD, 2021). While the declared trust in governmental institutions—relatively higher among Italian citizens—was not followed by greater compliance with the imposed measures, it may be associated with higher acceptability (and consequently lower stress levels) in those who pursued the local restrictions.

Both in the case of residents of Poland and Italy, the higher stress level was correlated with worse financial, occupational, and health situation—variables that are ubiquitously expected to be the most impacted by the development of pandemic (Heanoy et al., 2021). In both countries, stress was more prevalent among subjects outside a committed relationship, which may be associated with anxiety of not being able to expect any assistance in case of falling ill. However, people living in larger households were also prone to stress, perhaps by acknowledging the greater risk of disease dissemination among their close ones (Heanoy et al., 2021), by recognizing the family as a possible source of contagious disease transfer, or by simply feeling distressed because of forced isolation with a large group of people in a single household.

In Italy, residents of larger settlements had higher PSS scores than those living in small towns and in rural areas, while in Poland the highest level of stress was reported by people living in towns up to 150,000 inhabitants. These results may be a derivative to different fears among populations of these two countries. At the time of this study, Italy was facing a rapid increase of new cases and COVID-19-related deaths (Dong et al., 2020). The spread of the disease was causing a greater threat to people living in larger communities. On the other hand, Poland with few COVID-19 cases was struggling mainly with consequences of national quarantine, leading to financial instability and increasing unemployment (Główny Urza̧d Statystyczny, 2021) and deprivation of social contacts. In this case, living in a larger city, with greater perspectives of finding work and larger chances of sustaining social life, could be a reassuring circumstance. Moreover, people living in rural areas of Poland have stronger social ties and usually work in agriculture, a sector with comparably stable employment (RynekPracy.org, 2021).

The lowest PSS scores were observed in older, less educated respondents, in those who resided with only one other person, and in people who continued with their jobs on-site without shifting to remote work. Age seems to be a general protective factor for stress during pandemic (Bidzan-Bluma et al., 2020; Ruiz et al., 2021), as may be the lack of education in a new situation that requires analytical and interpretational skills to assess potential threats, although dominating evidence is to the contrary (Heanoy et al., 2021). Keeping one’s job intact while maintaining social contacts at work is also suggested to be protective against stress (Heanoy et al., 2021).

### Limitations

We recognize several limitations to this study, such as adopting the form of an open internet survey and its cross-sectional characteristics. Due to the difficulties associated with the first wave of pandemic and imposition of strict lockdowns in both countries, we adopted a form of convenience sampling with virtual snowball features with all their reliability limitations. Trying to minimize the sampling bias, various ways of reaching respondents were used (as described in Section 2.1) with the goal of achieving the most numerous samples possible, representing all relevant sociodemographic subgroups. At the end of May and at the beginning of June of 2020, the most significant lockdown restrictions were being lifted, which forced us to end the recruitment and therefore limit the number of responses. The responsiveness rates were different in Poland and Italy within the two-month period, resulting in uneven study groups. Although statistical methods were adopted to limit this distinction, one should be careful when generalizing the results with regard to the population in both countries and when interpreting the comparative differences. This is especially true for the Italian residents’ group, where the lower number of respondents, despite the greater total population, significantly limits the representativeness of samples.

The data were collected while both countries were facing a distinct pandemic course, with the first cases in Italy being reported over 1 month earlier than in Poland (Supplementary Figures 1, 2). Furthermore, there are several significant sociodemographic differences between the two groups (Table 1), with a large overrepresentation of females among respondents of both countries. No information on pre-pandemic perceived stress differences between samples of Polish and Italian residents is available, which will enable the comparison of the lockdown levels of stress to a baseline.

Moreover, we failed to fully identify the actual employment status of our respondents by not pursuing distinction between the long-term unemployed and those who had lost their jobs because of the imposed restrictions, which may have a significant effect on the perceived stress (Heanoy et al., 2021). Specific occupation of study participants was not evaluated. Apart from a brief self-reported distinction between pessimism and optimism traits, attitudes that are postulated to play a role in the development of stress reactions (Schou-Bredal et al., 2021), no other individual personality traits or states were analyzed in this study. While Ranieri et al. (2020) suggested that personality dimensions do not actually mediate distress or increase the risk for post-traumatic stress disorder, it seems that at least in certain groups, self-efficacy is a protective factor against stress during the pandemic (Bidzan et al., 2020).

Comorbidities were assessed only with regard to their presence; no data on specific conditions were gathered. Severe somatic condition or disability of respondents or among their families (over 90% of the subjects were worried about the health of their close ones) seem to impact the process of psychological and social adaptations to the pandemic (Khasawneh, 2020). Finally, we evaluated the respondents only with regard to their place of residence, not nationality nor migration status—factors that may also potentially play a role in stress adaptation mechanisms (Lanzara et al., 2019).

### Future Outlook

Possible directions for future studies involve exploring the data we omitted as described in the “Limitations” section. Comparison of stress levels of Polish and Italian residents and coping mechanisms in the coming months and years of the pandemic may also shed light on the possible explanation of the differences observed in this study. Further research comparing countries and/or regions in a similar stage of the pandemic may allow the elimination of some of the confounding factors.

## Conclusion

Despite several limitations to this study, significant differences between Polish and Italian residents in perceived stress level and compliance with lockdown policies suggest that we should be careful in overgeneralizing the impact of the pandemic and social isolation. While residents of some countries and world regions seem to adapt easily to various restrictions, simple copy–paste strategies may not be that beneficial. Potential short- and long-term effects of prolonged stress due to social isolation may eventually lead not only to severe mental and somatic health consequences but also ultimately to omnipresent non-compliance and movements such as the Polish “entrepreneur’s rebellion.”^4^Furthermore, a thorough look at the perceived consistency of pandemic policy measures may reveal their possible impact on the efficiency of social isolation and anxiety levels observed in the two countries. Radical and coherent state intervention significantly helped to contain the virus in China and other East Asia countries, but at the same time, this efficient solution could not be replicated with success in other regions of the world. While absolutely not discouraging governments from taking multiple preventive measures to stop the spread of the COVID-19 pandemic, we rather suggest that a tailor-made policy in each country may be more beneficial than a simple replication of solutions from other regions of the world.

## Data Availability Statement

The raw data supporting the conclusions of this article will be made available by the authors, without undue reservation.

## Ethics Statement

Ethical review and approval was not required for the study on human participants in accordance with the local legislation and institutional requirements. The patients/participants provided their written informed consent to participate in this study.

## Author Contributions

JS, TM, PW, MG, and JG contributed to conception and design of the study. AM, JR, RM, LL, and JG translated the survey. JG selected the data for analysis, created tables, and wrote the first draft of the manuscript. PW and JS did the statistical analysis. PW and JG created figures. JS, AM, RM, and JG created Supplementary Material. AM, JR, and JG did literature search. All authors distributed the survey and contributed to manuscript revision, read, and approved the submitted version.

## Conflict of Interest

The authors declare that the research was conducted in the absence of any commercial or financial relationships that could be construed as a potential conflict of interest.

## Footnotes

1 Europe is the new epicenter of coronavirus pandemic: WHO. Accessed January 26, 2021. https://nypost.com/2020/03/13/who-says-europe-is-new-epicenter-of-coronavirus-pandemic

2 Italy records its deadliest day of coronavirus outbreak with 475 deaths | World news | The Guardian. Accessed January 26, 2021. https://www.theguardian.com/world/2020/mar/18/coronavirus-lockdown-eu-belgium-germany-adopt-measures

3 https://ourworldindata.org/covid-mobility-trends

4 Spontaneous movement in Poland opposing government restrictions. Restaurants, nightclubs, gyms, and other facilities were opened despite interdiction by either using legal legerdemains or directly breaking the law.
