# Supplementary figures and images for: Social Isolation During COVID-19 Pandemic. Perceived Stress and Containment Measures Compliance Among Polish and Italian Residents
Source: Front Psychol. 2021 May 28;12:673514. doi: 10.3389/fpsyg.2021.673514 (PMC8194265; doi:10.3389/fpsyg.2021.673514)

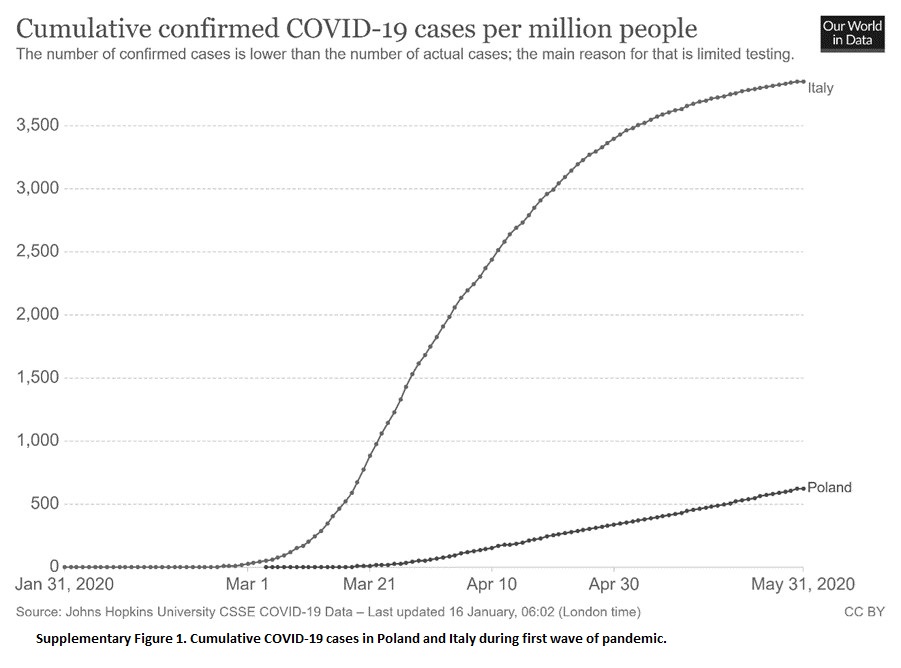

Supplement: Supplementary file 1 [file Image_1.JPEG]

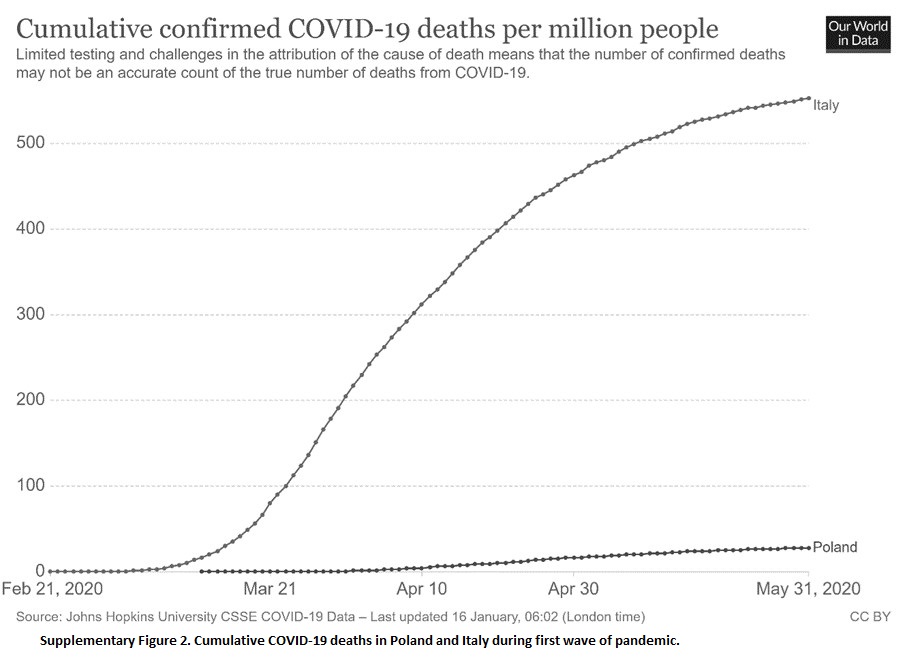

Supplement: Supplementary file 2 [file Image_2.JPEG]
