# Supplementary material for: Social Isolation During COVID-19 Pandemic. Perceived Stress and Containment Measures Compliance Among Polish and Italian Residents
Source: Front Psychol. 2021 May 28;12:673514. doi: 10.3389/fpsyg.2021.673514 (PMC8194265; doi:10.3389/fpsyg.2021.673514)
Supplement: Supplementary file 3 [file Table_1.DOCX]

Suppl. Table 1 Public health interventions to mitigate the early spread of SARS-CoV-2 in Poland (as of May 31, 2020).

| Type of measure | Details | Date enacted | In force |
| --- | --- | --- | --- |
| Crisis act: the Law on special arrangements for the prevention and combating of COVID-19, other infectious diseases and crisis situations caused by them (Ustawa..., 2020) | - Administrative, budgetary and epidemiological measures adapted to manage a possible epidemic of COVID-19 or other infectious diseases | March 2 | March 8 |
| Obligation to quarantine, epidemiological supervision or hospitalization related to COVID-19 | - People suffering from or suspected to have a COVID-19 can be ordered to undergo treatment in hospitals - Obligation to quarantine or epidemiological supervision persons who have close contact with confirmed or suspected COVID-19 cases | March 7 | March 7 |
| Border sanitary control | - Passengers’ Location Cards - Temperature measurement | March 9 | March 9 |
| Cancellation of mass events | - All mass events gathering at least 1,000 participants outside of buildings were cancelled - All events gathering 500 or more participants inside buildings were cancelled | March 10 | March 10 |
| Closure of universities | - All academic classes were cancelled - Researcher and scientific activities have been maintained, but scientists were encouraged to work remotely | March 11 | March 12 |
| Closure of schools and childcare providers | - Closure of all public and private educational institutions - All childcare providers were closed | March 11 | March 12 |
| Closure of culture institutions | - Cultural institutions, such as philharmonic orchestras, operas, theatres, museums, and cinemas suspended their activities | March 11 | March 12 |
| Unified hospitals for infectious diseases (COVID-19 hospitals) | - 19 hospitals were transformed into unified hospitals for infectious diseases, to provide full access to medical services for COVID-19 cases (at least one hospital located in each of the 16 administrative regions in Poland) | March 13 | March 16 |
| State of epidemic emergency | - Restaurants, bars, and cafes were able to operate only for takeaways and delivery - In shopping malls with a sales area of over 2,000 square meters, only grocery stores, pharmacies and laundry facilities remained open - Fairs, exhibitions, congresses, conferences and sporting events were suspended - Entertainment and recreation facilities including dance clubs, music clubs were closed - Public gatherings of more than 50 people were banned - Export of personal protective equipment and disinfection agents were limited | March 13 | March 14 |
| Temporary closure of borders to non-citizens | - Controls at all Polish borders were introduced - A ban on entry to Poland for foreigners (with some exemptions) was implemented - All citizens returning from abroad were obligated to undergo a 14-day civil quarantine - Compliance with the quarantine obligation was checked by the police and dedicated mobile application (“home quarantine”) - All international passenger air and rail services were suspended - A charter flight program called “Flight home” (in Polish “LOT do domu”) was implemented for Poles wishing to return from abroad | March 13 | March 15 |
| State of epidemic | - Organizational improvements to facilitate the prevention and combating of COVID-19 epidemics have been introduced | March 20 | March 20 |
| State of epidemic and its amendment | - Public gatherings were limited to a maximum of 2 people - Audience at the religious gatherings, funerals or marriages was limited up to 5 participants - People traveling on foot were obligated to keep a 1.5-meter distance - All non-essential travel was prohibited, with the exception of travelling to work, SARS-CoV-2 control related activities, or necessary everyday activities - Number of people who can use public transport was limited by half the capacity per vehicle | March 24 | March 25 |
| State of epidemic and its amendment | - Underaged were not allowed in public unattended - Forests, beaches and boulevards were excluded from use - Hairdresser’s and beauty, piercing, tattoo studios were closed - Hotels allowed to accommodate business trip guests only - Social distance between people extended to 2 meters(excluding child and handicapped carers) | March 31 | March 31 |
| Border sanitary control and epidemic amendment | - International transportation closure extended, - All newcomers were obligated to undergo a 14-day civil quarantine - Cultural institutions closed, - Amount of clients in commercial facilities set to 1 person/10 m^2^ | April 9 | April 9 |
| State of epidemic and its amendment | - Audience at the religious gatherings, funerals or marriages was extended to 50 participants | April 12 | April 12 |
| State of epidemic | - Law obligation to cover nose and mouth in public | April 16 | April 16 |
| State of epidemic and its amendment | - Amount of customers in commercial facilities extended to 1 person/15m^2^ - Recreational travel ban lifted | April 20 | April 20 |
| State of epidemic and its amendment | - Shopping centers, libraries, museums, art galleries, hotels, nurseries and kindergartens opened if following sanitary recommendations | May 4 | May 4 |
| State of epidemic and its amendment | - Restaurants, bars and cafes were opened if following sanitary recommendations as well as Hairdresser’s and beauty, piercing, tattoo studios | May 18 | Mat 18 |
| State of epidemic and its amendment | - Customers amount in commercial facilities regulations were lifted, - Audience at the religious gatherings, funerals or marriages was extended to 150 participants | May 30 | May 30 |

Based on Pinkas, Jankowski, Szumowski, Lusawa, Zgliczyński, Raciborski, Wierzba, & Gujski, 2020; updated by authors
