# Supplementary material for: Social Isolation During COVID-19 Pandemic. Perceived Stress and Containment Measures Compliance Among Polish and Italian Residents
Source: Front Psychol. 2021 May 28;12:673514. doi: 10.3389/fpsyg.2021.673514 (PMC8194265; doi:10.3389/fpsyg.2021.673514)
Supplement: Supplementary file 4 [file Table_2.DOCX]

**Table 2.** Public health interventions to mitigate the early spread of SARS-CoV-2 in Italy (as of June 3, 2020).

| Type of measure | | | | | Details | Date | In force |  |
| --- | --- | --- | --- | --- | --- | --- | --- | --- |
|  |  |  |  |  |  | enacted |  |  |
|  |  |  |  |  |  |  |  |  |
|  | | |  |  |  |  |  |  |
| Flight suspension | | |  |  | • Flights to and from China are suspended | 30 January | 30 January |  |
|  |  |  |  |  | • Announcement of the first two cases imported from China (Chinese tourists) | 2020 | 2020 |  |
|  |  |  |  |  |  |  |  |  |
|  | | |  |  |  |  |  |  |
| State of emergency | | |  |  | • Deliberation of the state of emergency for six months | 31 January | 31 January |  |
|  | | | |  |  |  |  |  |
| Repatriation procedures | | | |  | • Creation of a special unit for the repatriation of Italian | 1 February | 1 February |  |
|  |  |  |  |  | citizens located in China |  |  |  |
|  |  |  |  |  |  |  |  |  |
| Ministry |  | of |  | Health | • Compulsory isolation measures (quarantine) for close | 21 | 21 |  |
| Communique on quarantine | | | | | contacts with a positive patient | February | February |  |
|  |  |  |  |  | • Trustworthy isolation for those coming from risk |  |  |  |
|  |  |  |  |  | areas over the last 14 days and mandatory self- |  |  |  |
|  |  |  |  |  | notification to health authorities |  |  |  |
|  |  |  | |  |  |  |  |  |
| Decree | Law | (DL) | | and | Specific measures for certain municipalities located in | 23 | 23 |  |
| Presidential Decree | | | (DPCM): | | Lombardy and Veneto (“red areas”): | February | February |  |
| first | regional | | lockdown | | • Ban on moving from and to the municipality |  |  |  |
| measures | |  |  |  | • Ban on public and private events, including religious |  |  |  |
|  |  |  |  |  | celebrations |  |  |  |
|  |  |  |  |  | • Closing of every school and university, including ban |  |  |  |
|  |  |  |  |  | on school trips, as well as museums and other cultural |  |  |  |
|  |  |  |  |  | inistitutes, public offices, commercial activities |  |  |  |
|  |  |  |  |  | (excluding essential services) |  |  |  |
|  |  |  |  |  | • Mandatory use of individual protection devices when |  |  |  |
|  |  |  |  |  | entering essential services |  |  |  |
|  |  |  |  |  | • Suspension of public and private transportation |  |  |  |
|  |  |  |  |  | systems |  |  |  |
|  |  |  |  |  | • Implementation of smart working |  |  |  |
|  | | | | |  |  |  |  |
| DPCM on extension of regional | | | | | • Extension of restrictive measures for municipalities | 25 | 25 |  |
| lockdown measures | | |  |  | included in the decrees dated 23 February to sport | February | February |  |
|  |  |  |  |  | activities, tourism, health measures for prisons |  |  |  |
|  | | | | |  |  |  |  |
| DPCM on extension of regional | | | | | • Further extension of restrictive measures for “red | 1 March | 1 March |  |
| lockdown measures | | |  |  | areas” |  |  |  |
|  |  |  |  |  | • Implementation of uniformed prophylaxis measures |  |  |  |
|  |  |  |  |  | on a national level |  |  |  |
|  |  |  |  | |  |  |  |  |
| DPCM | on | first | national | | On a national level: | 4 March | 5 March |  |
| restrictions | |  |  |  | • Suspension of social events and meetings involving |  |  |  |
|  |  |  |  |  | healthcare or essential services workers |  |  |  |
|  |  |  |  |  | • Suspension of public and private events that cannot |  |  |  |
|  |  |  |  |  | guarantee the interpersonal distance of 1m |  |  |  |
|  |  |  |  |  | • Implementation of a closed-door policy for sport |  |  |  |
|  |  |  |  |  | events |  |  |  |
|  |  |  |  |  |  |  |  |  |

|  | • Closing of schools and universities and | |  |  |
| --- | --- | --- | --- | --- |
|  |  | implementation of remote teaching systems |  |  |
|  | • Ban on visits to hospitals and retirement homes | |  |  |
|  |  |  |  |  |
| DPCM on extension of regional |  |  | 8 March | 8 March |
| and national measures (replaced the decrees dated 1 and 4 March) | • | Creation of a unified “red area” including Lombardy, |  |  |
|  |  | Veneto, Emilia-Romagna, Piedmont and Marche |  |  |
|  |  | region, to be subjected to measures described above |  |  |
|  | • Introduction of further prevention measures on a national | |  |  |
|  |  | level. Suspension of sport events, closing of cinemas |  |  |
|  |  | and theaters, bingo rooms, clubs, pubs. |  |  |
|  |  | Implementation of preventive measures in |  |  |
|  |  | restaurants, churches and shops. Extension of schools |  |  |
|  |  | and universities closing. |  |  |
|  |  |  |  |  |
| DPCM on full lockdown | • | Extension of the “red area” to the whole national | 9 March | 9 March |
| measures |  | territory (see above). Lockdown until 3 April |  |  |
|  |  |  |  |  |
| DPC on extension of restrictive | • | Closing of all commercial shop except for essential | 11 March | 11 March |
| measures |  | services |  |  |
|  |  |  |  |  |
| DL “CuraItalia” (HealingItaly) | • | Economic measures for families, workers and | 16 March | 16 March |
|  |  | entrepreneurs |  |  |
|  |  |  |  |  |
| Creation of a task force | • | Creation of a task force of Medical Doctors to be | 19 March | 19 March |
|  |  | deployed to the most critical areas |  |  |
|  |  |  |  |  |
| Ministry of Health ordinance | • | Closing of parks, limitation of physical activities | 20 March | 21 March |
| on restrictions |  | within the immediate proximity of one’s home, |  |  |
|  |  | closing of all activities selling food and beverage |  |  |
|  |  | located in train and gas stations, ban on moving |  |  |
|  |  | towards holiday houses. |  |  |
|  |  |  |  |  |
| DL on discretionary restrictions | • | Introduction of emergency measures to control the | 24 March | 24 March |
| for specific areas |  | spread of the epidemic in specific areas, to be |  |  |
|  |  | implemented and modulated in time and content |  |  |
|  |  | according to the local situation |  |  |
|  |  |  |  |  |
| DPCM on economic measures | • | Supply of solidarity funds | 28 March | 28 March |
|  |  |  |  |  |
| DPCM on extension of | • | Extension of the lockdown until 13 April | 1 April | 1 April |
| lockdown |  |  |  |  |
|  |  |  |  |  |
| DL on economic measures | • | Economic measures | 6 April | 6 April |
|  |  |  |  |  |
| DPCM on extension of | • | Extension of the lockdown until 3 May | 10 April | 14 April |
| lockdown and first relaxation | • | Opening of stationery stores, bookshops and shops |  |  |
| of measures |  | for children and infants |  |  |
|  |  |  |  |  |
| Council of Ministers on the | • | Allocation of further €900.000.000 for medical | 20 April | 20 April |
| budget allocation for the Covid |  | equipment |  |  |
| Task Force |  |  |  |  |
|  |  |  |  |  |
| DPCM on rolling out of “Phase | • | Price control on masks | 26 April | 27 April |
| 2” | • | Opening of public parks |  |  |
|  | • | Mandatory mask use |  |  |
|  |  |  |  |  |

|  |  |  | • Allowing for food collection from restaurants |  |  |  |  |
| --- | --- | --- | --- | --- | --- | --- | --- |
|  |  |  | • Gradual opening non-essential enterprises starting |  |  |  |  |
|  |  |  | from 4 May |  |  |  |  |
|  |  |  | • Allowing for indoor training |  |  |  |  |
|  |  |  | • Allocation of financial aids for families, workers and |  |  |  |  |
|  |  |  | enterprises |  |  |  |  |
|  | | |  |  |  |  |  |
| State-ChurchProtocolon | | | • Restart of religious celebration with all health | 7 | May | 18 | May |
| restart of religious celebration | | | precautions |  |  |  |  |
|  |  |  |  |  |  |  |  |
| DL on | a | seroprevalence | • Carrying out of a seroprevalence analysis on a | 9 | May | 9 | May |
| analysis |  |  | national scale |  |  |  |  |
|  | |  |  |  |  |  |  |
| DL “Rilancio” | |  | • Financial measures for the reopening of non-essential | 13 | May | 13 | May |
|  |  |  | activities |  |  |  |  |
|  | | |  |  |  |  |  |
| DL on preventive measures | | | • Legal framework to allow for national, regional and | 15 | May | 16 | May |
|  |  |  | local regulations for limitations on movements and |  |  |  |  |
|  |  |  | activities within specific territories |  |  |  |  |
|  |  |  |  |  |  |  |  |
| Reopening | of | interregional | • Reopening of regional borders allowing for | 3 | June | 3 | June |
| movement |  |  | interregional movement of people |  |  |  |  |
|  |  |  |  |  |  |  |  |

Source: [Italian Government](http://www.governo.it/it/coronavirus-misure-del-governo)
